# EEG Spectral Features Discriminate between Alzheimer’s and Vascular Dementia

**DOI:** 10.3389/fneur.2015.00025

**Published:** 2015-02-13

**Authors:** Emanuel Neto, Elena A. Allen, Harald Aurlien, Helge Nordby, Tom Eichele

**Affiliations:** ^1^Institute of Biological and Medical Psychology, University of Bergen, Bergen, Norway; ^2^Section for Clinical Neurophysiology, Haukeland University Hospital, Bergen, Norway; ^3^K. G. Jebsen Center for Research on Neuropsychiatric Disorders, Bergen, Norway; ^4^The Mind Research Network, Albuquerque, NM, USA

**Keywords:** Alzheimer’s disease, vascular dementia, electroencephalogram, qEEG, quantitative analysis

## Abstract

Alzheimer’s disease (AD) and vascular dementia (VaD) present with similar clinical symptoms of cognitive decline, but the underlying pathophysiological mechanisms differ. To determine whether clinical electroencephalography (EEG) can provide information relevant to discriminate between these diagnoses, we used quantitative EEG analysis to compare the spectra between non-medicated patients with AD (*n* = 77) and VaD (*n* = 77) and healthy elderly normal controls (NC) (*n* = 77). We use curve-fitting with a combination of a power loss and Gaussian function to model the averaged resting-state spectra of each EEG channel extracting six parameters. We assessed the performance of our model and tested the extracted parameters for group differentiation. We performed regression analysis in a multivariate analysis of covariance with group, age, gender, and number of epochs as predictors and further explored the topographical group differences with pair-wise contrasts. Significant topographical differences between the groups were found in several of the extracted features. Both AD and VaD groups showed increased delta power when compared to NC, whereas the AD patients showed a decrease in alpha power for occipital and temporal regions when compared with NC. The VaD patients had higher alpha power than NC and AD. The AD and VaD groups showed slowing of the alpha rhythm. Variability of the alpha frequency was wider for both AD and VaD groups. There was a general decrease in beta power for both AD and VaD. The proposed model is useful to parameterize spectra, which allowed extracting relevant clinical EEG key features that move toward simple and interpretable diagnostic criteria.

## Introduction

Alzheimer’s disease (AD) is a debilitating neuro-degenerative disease, and is one of the most common forms of dementia among the elderly population (1) with a significant socio-economic burden for societies in developed countries. AD particularly affects individuals over the age of 65 years and it is estimated that its prevalence will triple within the next 40 years (2, 3). Vascular dementia (VaD) may result either from ischemic or hemorrhagic cerebrovascular disease (CVD), or from cardiovascular or circulatory disturbances that injure brain regions relevant to memory, cognition, and behavior (4). VaD is the second most common form of dementia after AD, affecting approximately 20% of the dementia cases worldwide (5). While VaD can be assessed with the use of imaging techniques at early stages of the disease, the similarities between symptoms between the different conditions can lead to diagnostic uncertainty. Autopsy assessment studies in dementia report that VaD was present in 24–28% of AD cases (6, 7). One current difficulty is the relative lack of specific biomarkers; certain diagnosis for AD is still only possible through a *post-mortem* exam; routinely several examinations, such as the mini-mental state examination (MMSE), positron emission tomography (PET), computer tomography (CT), magnetic resonance imaging (MRI), and electroencephalogram (EEG) are used during differential diagnostics (8, 9). So far, clinical EEG assessment is mainly performed through visual inspection, identifying clear signs of pathology, while usually not considering other quantifiable measures. Potentially relevant features that are not immediately visible, such as power modulations, connectivity changes, or sparse small amplitude phenomena, may thus be overlooked.

Quantitative EEG analysis therefore may be helpful in the clinical context. For example, it is well known that decreases of alpha and beta power and increases of the delta and theta frequencies are related to brain pathology and general cognitive decline (10–13). Recent studies have demonstrated that AD has a pre-symptomatic phase that can last for years, known as mild cognitive impairment (MCI) and while neuronal degeneration is taking place, the clinical symptoms remain subtle. Consequently, early behavioral and pharmacological interventions, which can ameliorate the course of the disease, should not be administered based on clinical data alone (14–18). However, abundant literature reports that specific drugs induce alterations on electroencephalographic readings. A comprehensive overview of recent studies clusters typical effects with different pharmacological agents (19). Specifically, drugs that act on the nervous system such as psycholeptics and psychoanaleptics may induce neuronal hyperexcitability or drowsiness and hence EEG patterns change (20). Nevertheless, it has been shown that the resting EEG activity can predict future cognitive decline or conversion to dementia in MCI subjects with high accuracy (21–25). Furthermore, recent studies suggest that spectral analysis can be used to distinguish AD from other dementias (26–29). These studies use various EEG markers such as spectral power, coherence, and frequency of rhythms in delta, theta, alpha, or beta bands, which are considered valuable markers for group classification by several studies (11, 30–35). However, the more EEG features studied, the larger sample sizes are required, which is often not easy to obtain. Moreover, many EEG studies using qEEG analysis for classification of AD differ on the test-paradigm, sample size, methods, features extracted, and classification models (36). The implementation of systematic guidelines to access dementia through the use of core EEG markers would facilitate these methods and provide more material for research.

In an effort to meet the needs of clinical EEG community, this study proposes a model for extracting EEG spectral features in groups of dementia patients, which might also be used as biomarkers for other diseases involving encephalopathy. This study shows the potential to differentiate between two of the most common types of dementias (AD and VaD) at the group level. In particular, we performed quantitative spectral analysis on clinical EEGs collected from a large in-house database (37), which holds over 30,000 EEG records. We applied a curve-fitting algorithm to model the frequency spectrum of each patient, extracting a total of six parameters from each channel. These features represent low (delta) and high (beta) frequency bands, decay of amplitude from low to higher frequencies, alpha power, alpha frequency, and dispersion of alpha.

## Materials and Methods

### Design

We performed a retrospective analysis of AD and VaD patients, examining EEGs that were collected as part of clinical diagnostic procedure.

### Sample

The database of the neurophysiology department of Haukeland University Hospital contains more than 37,000 EEG datasets from about 23,000 subjects available internally for research. From this database, we initially selected a convenience sample of all datasets from outpatients diagnosed with AD (*n* = 534), with international classification disease (ICD) codes ICD-10, F00.x and G30.x; VaD (*n* = 203) (ICD-10 F01.X), and NC (*n* = 3138) of non-hospitalized individuals at the time of the EEG recording, free of medication, and with no brain disease on record. The mean age and SD of the initial sample were 74.34 ± 9.92, 72.41 ± 11.16, and 52.2 ± 14.38 years for AD, VaD, and NC, respectively. We applied stringent criteria for inclusion and exclusion of samples as described in Sections “Selection Criteria” and “Pre-processing,” remaining with a total of 242, 88, and 1950 datasets on AD, VaD, and NC groups, respectively. The samples were then age and gender matched, leaving 77 cases in each group, with an average age of 73.1 ± 10.4 years and 51% males.

#### Selection criteria

For the initial sample described above, we applied the same inclusion criteria to all groups. First, we excluded patients with ages below 35 years old at the time of the recording. Second, we excluded the EEG recordings performed when subjects were hospitalized. Lastly, we excluded multiple EEG datasets from each patient including only the latest EEG dataset from each.

A general exclusion rule was applied when incongruent, insufficient, or poor data quality was found on the patient’s clinical or personal information, which excluded 988 patients. We considered the medications prescribed to each patient at the time of EEG recording, and excluded patients who were taking anticholinergic and dopaminergic agents, antipsychotics, anxiolytics, hypnotics and sedatives, anti-depressants, and psychostimulants. A complete list of the medications prescribed was resumed in Table S1 in Supplementary Material. The entire clinical diagnose history available from each patient’s record was taken into consideration: for the patients belonging to NC, we excluded those who had been diagnosed at any time point (prior or post the EEG recording date) with any CVD, any mental, behavioral, or neurodevelopmental disorders. For the AD and VaD groups, we excluded those who had been diagnosed at any time with any other brain or CNS-related disorder. In particular, the AD and VaD groups contained datasets from patients who had been only diagnosed with Alzheimer disease or VaD, respectively, before or persistently after the EEG recording date. For further details on the selection criteria, please see Table S2 in Supplementary Material. From the initial sample, we considered 2092, 269, and 118 datasets for NC, AD, and VaD groups, respectively, which were submitted to the pre-processing. A total of 199 datasets were excluded during the pre-processing due to insufficient epochs (see next section). The final sample was constituted by 77 age- and gender-matched subjects on each group with age averages of 71.5 ± 11.2, 73.5 ± 9.9, and 74.1 ± 9.9, respectively. The gender distribution was 49, 51, and 52% males for NC, AD, and VaD groups, respectively.

#### Pre-processing

**Figure 1:**
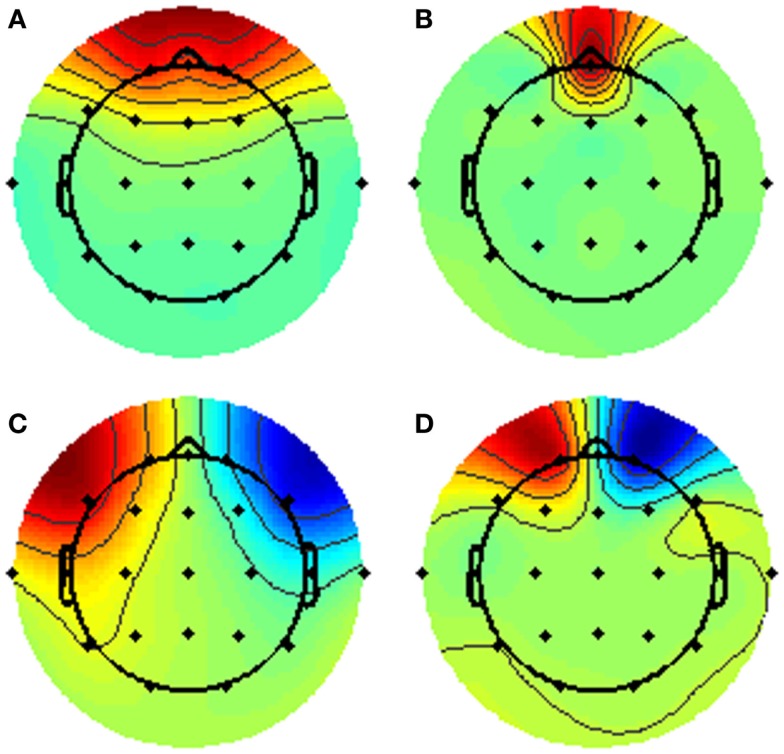
**Topographic templates of eye muscular artifacts used in ICA: eye blink – (A,B); lateral eye movement – (C,D)**.

**Figure 2:**
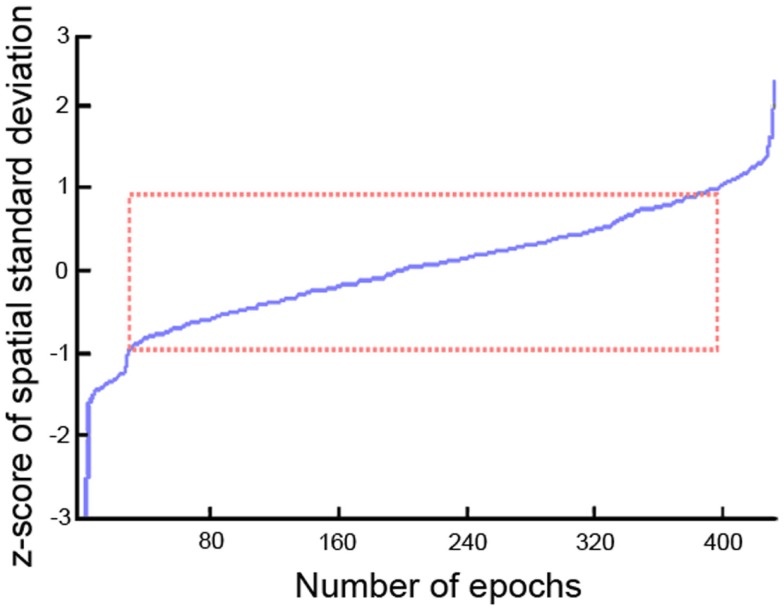
**Discrimination of epochs using the spatial standard deviation (sSTD) index**.

All EEG datasets were acquired using 22 channels positioned in 10–20 system placements (Fp1, Fpz, Fp2, F7, F3, Fz, F4, F8, T3/T7, C3, Cz, C4, T4/T8, T5/P7, P3, Pz, P4, T6/P8, O1, O2, M1, M2) acquired at 128 Hz (*n* = 59), 256 Hz (*n* = 164), and 500 Hz (*n* = 8) using NicoletOne™ EEG system. Input impedances were set to *Z* > 100 MΩ. Hardware single pole high-pass (0.16 Hz ± 10%) and low-pass (500 Hz ± 10%) filters were applied individually to each channel before pre-amplification. EEGs were stored under raw format in the database. All the pre-processing and data analysis were performed in the Mathworks^^®^^ Matlab environment. EEG raw files were imported to the EEGLab v.10.1.1.0b toolbox (38) using an in-house data-reader. Data were resampled to 256 Hz. From the standard clinical EEG recording protocol that lasts for 20 min and includes eye open/closed conditions, hyperventilation, and provocations with photic stimulation, we restricted the input data for analysis to the first 9 min, which contained only the alternating eyes open/closed resting conditions. A 1536-points high-pass-band filter was applied with cut-off frequency of 0.5 and a low-pass filter with cut-off of 50 Hz using a standard least square linear-phase FIR filter design. EEGs were segmented into non-overlapping epochs of 1 s that were evaluated for possible rejection using automatic amplitude, power, and statistical thresholding. The remaining segments were subjected to an individual independent component analysis (ICA) using the Infomax algorithm with 15 components in order to identify and remove residual contributions from eye movements. Four spatial templates for these types of artifacts were generated using 24 randomly drawn datasets, in which the corresponding artifact maps were visually identified and averaged across subjects to form the templates. Figure 1 displays the eye movement templates. The ICA topographies from each EEG dataset were correlated against the artifact templates and were removed if an absolute correlation coefficient above 0.8 was found. The continuous data were reconstructed from the non-artifact components and then segmented into 2 s epochs with 1 s overlap, which is equivalent to the Welch’s procedure (39) with a rectangular windows and 50% segment overlap. Subsequently, the data were transformed into the frequency domain using fast Fourier transform (FFT). Since the frequency spectrum selected for the pre-processing was from 0.5 to 50 Hz, we obtained 100 frequency data points for the 22 channels and a variable number of epochs for each dataset subjected for analysis. The spatial standard deviation (sSTD) index of each epoch was calculated across the 22 channels in the frequency domain according to (40) and *z*-scored. Epochs where the sSTD index exceeded |*z*| > 1 were excluded from further analysis, as illustrated in Figure 2. With the exclusion of such epochs, we ended up with a limited number of available segments of data for the initial sample. In order to unify the amount of data across subjects for the final sample, we determined the minimum common number of existing epochs across subjects that maximized the inclusion. This resulted in the selection of 334 epochs of data from each dataset (approx. 5 m 30 s). Subjects with fewer than 334 epochs were excluded from the initial sample. The available epochs of the final sample followed a normal distribution across the three groups with averages of 386 ± 24 for the NC group, 391 ± 28 epochs for AD, and 387 ± 20 for the VaD. No significant differences were found in the amount of available segments of data between the three groups (*F* = 1.03, *p* = 0.43). Epochs that contained higher amplitude in the range of the alpha band (8–12 Hz), were given priority for inclusion and remaining epochs were discarded. In this way, we expected to maximize the expression of the alpha frequency in the analysis. After age-gender matching of the groups, the final sample used in the analysis was composed of 334 epochs of data for each subject and 77 subjects in each group.

### Group spectrum differences

**Figure 3:**
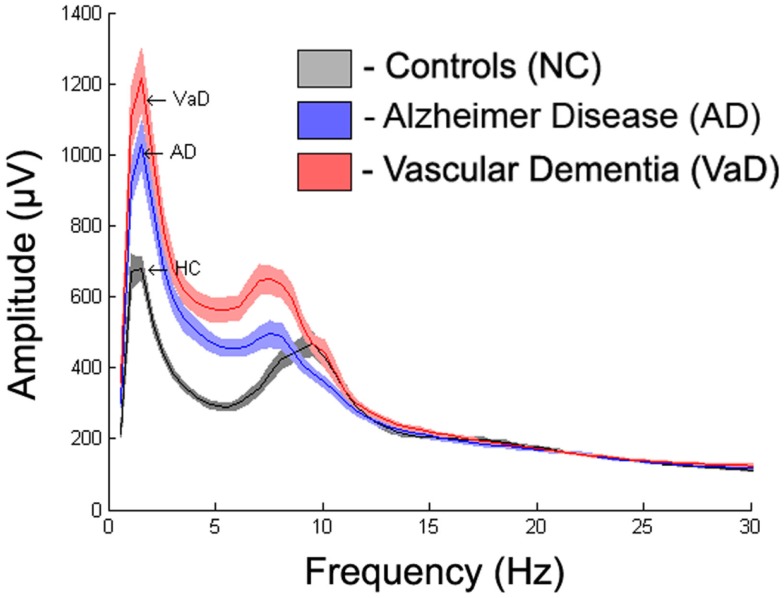
**Averaged frequency spectrum over all channels for each group with absolute frequency**.

To display the general differences between groups, we averaged the spectra first over all epochs within subjects, then over all subjects within each group. The mean spectrum ± 1 standard error of the mean (SEM) for each group are displayed in Figure 3. Further spectrum differences were explored using a curve-fitting model for comparison

### Curve fitting

To describe the power spectrum in a concise way, we combined a power-loss function modeling the roughly 1/*f* drop-off and a Gaussian function centered on the alpha-frequency peak. Specifically, we modeled the spectral power, *P*(*f*), with the following expression:

$$P\left(f\right)=Sf^{-k}+Ae^{-\frac{{\left(f-c\right)}^{2}}{w}}+b$$

**Figure 4:**
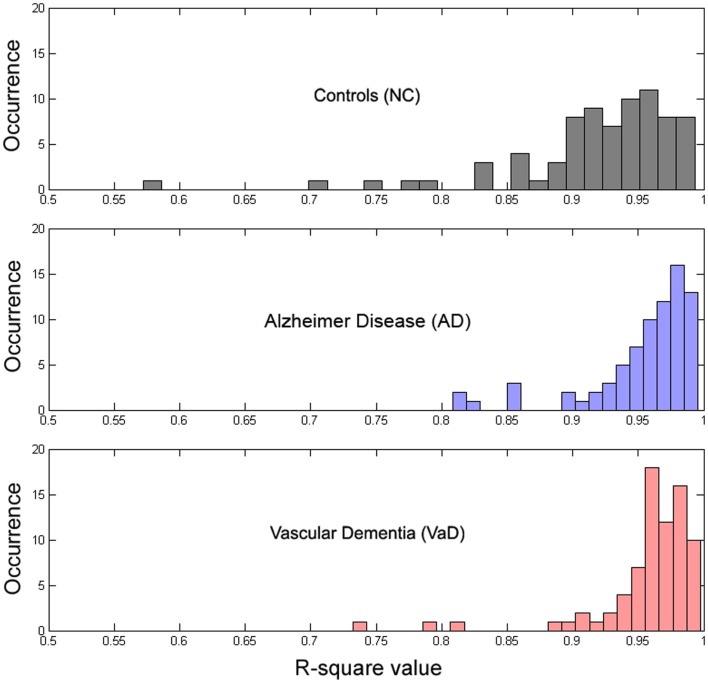
**Histogram of the distribution of R^2^ values for each group**.

**Figure 5:**
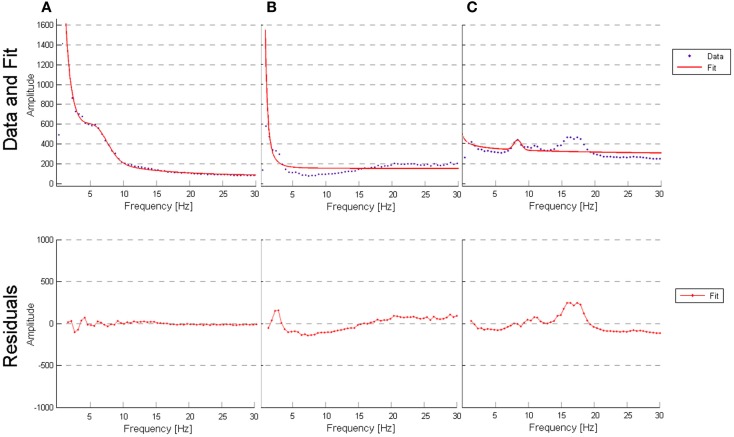
**(A)** Outlier for best fit performed by the model (AD patient, channel T6, *R*-square value of 0.9955); **(B)** outlier for worst fit performed by the model (VaD patient, channel Fp2, *R*-square value of 0.7324); **(C)** outlier for worst fit performed by the model (subject from NC, channel O1, *R*-square value of 0.5724).

All parameters were estimated using a non-linear least squares trust-region optimization algorithm, as provided in Matlab’s fit-curve toolbox. The model was fit to the power spectrum (averaged across epochs) in the frequency range of 1–30 Hz, to avoid tapering effects caused by the low-pass filter (0.5 Hz). We applied a particular exclusion rule for the fit of the power-loss function, excluding data points that correspond to the alpha band range (7–13 Hz). For the fitting curve algorithm, upper and lower estimation boundaries for each of the parameters were determined based on fits to the grand average spectra and are provided in Table 1. The *goodness-of-fit* of the model was assessed with the *R*^2^ statistic (Figure 4), and examples of best and worst fits are shown in Figure 5. The six parameters used in our model can be related to classic electrophysiological markers. Free parameters *S* and *k* relate to the power-loss function and represent its *scale* and *loss*, respectively. Values of *S* represent the amplitude at lower frequencies (delta waves), while *k* indicates the roughly 1/*f* decay of amplitude from lower to higher frequencies. Larger values of *k* denote a faster drop-off in power. Parameters *A*, *c*, and *w* relate to the Gaussian and represent the *amplitude*, *center*, and *dispersion* of the alpha peak, respectively. The alpha peak, here characterized by the amplitude, *A* and the center, *c*, which is often described in the literature as the occipital dominant rhythm (41–44) and is related to brain pathology and may potentially be used as reliable markers for a group differentiation (45, 46). Parameter *w*, which represents the dispersion of the alpha peak, has been described previously as the dominant frequency variability (47). Parameter *b* represents a global offset or *baseline* power of the entire frequency spectrum where the drop function and Gaussian best fit on and is related with the limit of the high frequency (beta) amplitude. For a general group comparison, we used the results from parameter *c* to estimate the averaged value of the alpha frequency for each group across all channels.

**Table 1:** **Fitting curve algorithm setup with initial, upper and lower boundaries for each of the six parameters**.

Parameter	Interpretation	Initial	Lower limit	Upper limit
*S*	Low frequency power	1000	0	∞
*k*	Decay from lower to higher frequencies	1	0	5
*A*	Alpha power	50	0	∞
*c*	Alpha frequency	8	6	14
*w*	Alpha dispersion	1	0.25	25
*b*	Baseline of the entire frequency spectrum	60	0	∞

### Statistical analysis

**Figure 6:**
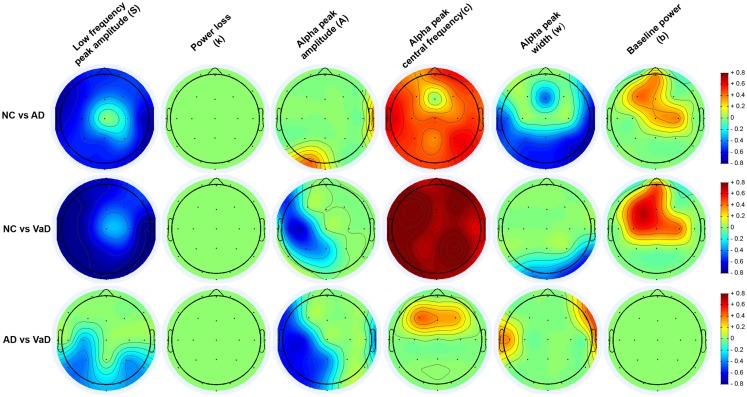
**Topography of the parameters effect sizes (*S*, *k*, *A*, *c*, *w*, and *b*) between groups (NC, AD, and VaD)**. Red or blue color gradients denote, respectively, a positive or negative effect when comparing the groups using Cohen’s *d* effect size scale (|*d*| < 0.2 – small effect; 0.2 < |*d*| < 0.8 – medium effect; |*d*| > 0.8 – large effect).

Because of the mild covariance between some spectral parameters (see Results), we used a multivariate analysis of covariance (MANCOVA) model as implemented in the MANCOVAN toolbox (42) to understand group differences while controlling for other nuisance factors. Prior to modeling, we used Box–Cox power transforms to improve normality of parameters *S* and *A*, which were highly skewed. Lambda values (Box–Cox parameters) were determined by maximizing the log-likelihood of normality and were 0.07 and 0.27, respectively. We first applied a MANCOVA to the spectral parameters averaged over all channels, and used a stepwise regression procedure with backward elimination to remove predictors that did not account for significant variance. Specifically, our initial model included the following predictors: group (an indicator variable coding for NC, AD, and VaD), age, gender, number of ICA components removed during pre-processing, original sampling-rate, and number of clean epochs initially available; our dependent variable was a [342 × 6] matrix, which contained the six spectral parameters (*S*, *k*, *A*, *c*, *w*, and *b*) averaged over channels for the 342 subjects. Following stepwise reduction with an alpha value of 0.01, the final model retained group (*T* = 76.23, *p* = 7.1 × 10^−10^), age (*T* = 34.79, *p* = 1.7 × 10^−5^), gender (*T* = 23.89, *p* = 1.0 × 10^−3^), and number of epochs (*T* = 38.7, *p* = 3.8 × 10^−6^) as highly significant predictors. We then used these predictors in a second MANCOVA to model the spectral parameters at each channels, i.e., the dependent variable was a [342 × 132] matrix, representing 6 parameters at 22 channels, for all subjects. We confirmed that the four predictors (group, age, gender, and number of epochs) still accounted for significant variance in this larger model (*p* < 0.005 for all terms). Significance of the “group” factor at the channel level was determined from the *F*-statistics and corresponding *p*-values as shown in the Section “Results.” *p*-Values were corrected for multiple comparisons using False Discovery Rate (FDR, *q* = 0.05). For channels/parameters meeting significance, pair-wise contrasts were performed between groups (i.e., NC vs. AD, NC vs. VaD, and AD vs. VaD). Pair-wise contrasts are denoted as significant at the (uncorrected) level of *p* < 0.05, very significant at *p* < 0.01, and highly significant at *p* < 0.001, highlighted in Figures S1–S6 in Supplementary Material with “*,” “**,” and “***,” respectively. For brevity, in presentation of our results, we determined the effect size for the group differences found at significant levels *p* < 0.05 using Cohen’s *d* (48) and displayed on topographical maps as shown in Figure 6.

## Results

### Group spectra

Figure 3 displays the average spectra across all channels for the NC, AD, and VaD groups. Clear group differences were observed in the delta, theta, and alpha-frequency bands. These differences were quantified by comparing the six parameters (*S*, *k*, *A*, *c*, *w*, and *b*) used in the model, as described in the section “Materials and Methods.”

### Evaluation of the model

To determine whether our model fit the data well, we calculated the *R*^2^ measure for each fit, reflecting the fraction of data variance captured by the model. Additionally, we computed the correlations between model parameters to check for dependence between parameters, which can indicate model redundancies or instabilities in the fitting procedure. Histograms of the *R*^2^ values are displayed in Figure 4, and show excellent model fits in nearly all cases. The median goodness-of-fit was 0.96 and the first and third quartiles were 0.92 and 0.98, respectively. We performed a Kruskal–Wallis one-way ANOVA test and no group differences were found in the *R*^2^ values between the groups (*p* < 3.5e^−8^), meaning that the model performed equally well fitting the datasets from each group. Examples of best and worst fits (as determined from the *R*^2^ values) are shown in Figure 5. Only relatively weak correlations (|*r*| ≤ 0.4) between the parameters were found, with the exception of a high correlation between parameters *k* and *b* (*r* = 0.74). This correlation was equally present within each group: *r* = 0.81 for NC, *r* = 0.64 for AD, and *r* = 0.79 for VaD.

### Group differences

Group differences in spectral parameters were determined from the MANCOVA applied separately at each channel for all six parameters. For brevity, we report only findings that passed the FDR correction for significance (see Materials and Methods). Figure 6 represents the topographical differences on a scale of Cohen’s *d* effect size, where |*d*| < 0.2 is considered a small effect; 0.2 < |*d*| < 0.8 medium effect; |*d*| > 0.8 – large effect. For full details, readers are referred to Figures S1–S6 in Supplementary Material, which display the average parameter values, *F*-statistics, and *p*-values for each channel and group comparisons.

#### [*S*] (low frequency power)

As shown in Figure 6, both AD and particularly VaD patients had larger *S* compared to NC. This difference was highly significant for most channels (*F* > 7, *p* < 1.52 × 10^−3^). The VaD group also showed significantly higher *S* than AD patients for parietal, temporal, and occipital channels (*F* > 17, *p* < 1.51 × 10^−7^). (*1/f* decay from lower to higher frequencies)]

#### [*k*] (*1/f* decay from lower to higher frequencies)

No significant differences were found between AD, VaD, and NC group at group level. (alpha power)]

#### [*A*] (alpha power)

Significant differences were observed in the VaD group compared with NC, with VaD showing larger *A* values over the left hemisphere and central channels (*F* > 4, *p* < 2.36 × 10^−2^). VaD also showed significantly greater *A* values compared with AD in nearly the same regions. AD, however, had significantly lower A than NC at occipital-left and lateral-right channels. (alpha frequency)]

#### [*c*] (alpha frequency)

Vascular dementia patients had significantly lower *c* values compared with NC for all channels (*F* > 8, *p* < 8.43 × 10^−4^). The same trend was observed for AD compared with the NC group, where AD patients had lower *c* values at all channels. At central and frontal channels, VaD showed lower *c* compared with AD. Mean *c* values at occipital channels, were 9.4, 8.4, and 8.1 Hz for NC, AD, and VaD patients, respectively. (alpha dispersion)]

#### [*w*] (alpha dispersion)

Higher *w* was found in AD compared with NC at central, occipital, and parietal areas (*F* > 4, *p* < 2.53 × 10^−2^), while VaD had higher *w* at occipital and right-temporal channels compared with NC (*F* > 7, *p* < 1.46 × 10^−3^) The AD group had significantly higher *w* than VaD mainly at lateral electrodes (*F* > 5, *p* < 1.65 × 10^−2^) (baseline of the entire frequency spectrum)]

#### [*b*] (baseline of the entire frequency spectrum)

For the parameter *b*, significant differences were found between NC and both AD and VaD groups. Both AD and VaD groups displayed significantly lower *b* than NC at central and frontal channels (*F* > 4, *p* < 2.5 × 10^−2^).

## Discussion

This study explored electrophysiological differences that might be used as markers to automatically distinguish between groups. In order to assess those differences, we implemented quantitative analysis in clinical EEGs of NC, AD, and VaD and applied a model that extracted six EEG parameters to describe the average spectrum from each channel. Preliminary results (Figure 3) indicated marked differences between the three groups. Our approach uses standard models with relatively simple implementation and low computational costs. The EEG features extracted by our model are related to key relevant EEG markers for clinical assessment. We tested our model in AD and VaD group differentiation using the extracted features. Significant differences were found for several of the parameters with topographical specificity between AD and VaD in relation to healthy subjects. Our results inspire the use of such model as a standard approach for the extraction of EEG features and possible future use of such features as biomarkers in group differentiation.

### Spectrum model

The curve-fitting approach allowed us to describe the frequency spectrum using only six parameters, which are related with known electrophysiological markers. In general, we found that the model is a good fit to the data most of the time and that performs equally well regardless of the type of participant (see “Evaluation of the model” in “Results” section). We observed that a poor fit occurred in two situations: (1) subjects/channels where the spectrum curves contained a clear and distinguished theta peak that was distinct from alpha; (2) subjects/channels where low beta frequency amplitude was pronounced (as seen in Figure 5). Additionally, a correlation between the six parameters verified that parameters *k* and *b* are highly correlated (*r* = 0.75). This correlation points to a weakness in the model, since it appears that parameters *k* and *b* may work against each other.

### Group differences

#### Patients vs. controls

Our results revealed significant increase of *S* and a decrease of *b* comparing the AD group with NC, in line with previous literature (49). Parameter *c* was 9.4 Hz in NC compared to 8.5 Hz in AD. Similar alpha slowing from 10.2 to 8.1 Hz has been reported previously (50). Our study also indicates that AD patients have lower *A* than NC. Slowing of alpha frequency and decreased alpha power have been reported in several studies, when comparing AD and also MCI patients with healthy controls (36, 50–55). This is thought to be associated with general cognitive decline (10–13). AD patients had higher *w* than NC, in agreement with the findings from (47). VaD also had increased *S*, lower *b*, and increased *A* compared with NC. The decrease of the *c* was 9.4 to 8.1 Hz when compared with NC. (50) reported the corresponding values as 10.2–8.3 Hz. In contrast to the AD group, VaD had higher *A* in occipital regions than NC, in accordance with previous studies (45, 56, 57). It is well established that the source of the alpha rhythm is clearly predominant at occipital cortical regions (58). Other studies suggest that for AD and VaD patients, the increase of the alpha power is positively correlated with the glucose metabolism in the occipital lobe whereas the increase at lower frequencies of the EEG is negatively correlated with metabolism (59).

#### VaD vs. AD

Our results show significant differences for the *S* parameter between VaD and AD, in particular, an increase of *S* for the VaD group for occipital, temporal, and parietal channels compared to the AD group, see Figures 3 and 6. In addition, VaD had significantly higher *A* when compared with AD at occipital, temporal, lateral, and frontal electrodes. Interestingly, we observe a more pronounced decrease in *c* for the VaD patients. Only very few studies have performed spectral comparisons between these two groups. Nevertheless, the slowing of alpha rhythm and the increase of power in the delta and theta band were more pronounced in VaD than in AD patients, as reported in the literature (50, 60). The general increase of *S* along with decrease in *b* and further slowing of *c* have been reported in other studies and was suggested the use of such markers for accessing cognitive function (10–13) in MCI and AD studies.

Parameter *w* differentiates patients with AD both from VaD and from NC at several channels. This parameter is similar to the dominant frequency variability (47), which was found to differentiate between AD, Lewy body, and Parkinson’s disease. It is known that occipital frequency differences persist both in AD and VaD patients (45). Furthermore, at early stages of the disease, the neuropsychological and behavioral tests provide subtle differences leading to miss diagnoses of AD in VaD patients or vice-versa (6, 7). Therefore, we believe particularly this parameter *w* to be an interesting biomarker for group differentiation.

### Limitations

As we discussed previously, the EEG datasets that we included in our study were collected during normal clinical routine from the hospital and we relied on the latest existing clinical working ICD-10 diagnosis determined by neurologists, (geronto-)psychiatrists, and neuropsychologists’ staff. Despite the stringent criteria used to select these patients, there is still an uncertainty on diagnosing VaD or AD, which can only be confirmed by a post-mortem test. More detailed reports of patient performance on various tests would require full access to patient data, which was not feasible here. Also, we have not correlated EEG findings with clinical scores such as MMSE, such that we cannot speculate about the relationships between severity of dementia symptoms and EEG abnormalities. Therefore, the group differences described in this study are biased to the uncertainty of diagnoses of our sample and should not *per se* be fully generalized.

## Conclusion

Since this study included a large sample of unmedicated, age, and gender matched of AD, VaD, and NC with clinical EEGs, it was possible to compare the spectral differences between these groups simultaneously. The model used in this study extracted relevant EEG features such as low and high frequency amplitudes, decay of amplitude from low to high frequencies, alpha frequency, alpha power, and dispersion of alpha frequency. The implementation of this model is suitable for analyzing EEG, and has relatively low time and processing costs by reducing the complexity of the broad spectrum curve. Our findings revealed significant differences in several of the features extracted by the model, namely lower alpha power at lateral-occipital regions for AD patients when compared with NC in contrast to VaD patients who had higher power compared with NC for the same regions. Both AD and VaD patients had higher delta power than NC and, for posterior channels. VaD patients had even higher delta power than AD patients. The dispersion of alpha, *w*, was a novel feature extracted by our fit-curve model and established quantifiable differences between AD and VaD. Based on this study, we believe that the proposed model is useful for extraction and quantification of differences between these groups of patients, and may have the potential to be used as a general tool in quantitative EEG analysis.

## Conflict of Interest Statement

The authors declare that the research was conducted in the absence of any commercial or financial relationships that could be construed as a potential conflict of interest.

## Supplementary Material

The Supplementary Material for this article can be found online at http://www.frontiersin.org/Journal/10.3389/fneur.2015.00025/abstract
