# Supplementary material for: EEG Spectral Features Discriminate between Alzheimer’s and Vascular Dementia
Source: Front Neurol. 2015 Feb 13;6:25. doi: 10.3389/fneur.2015.00025 (PMC4327579; doi:10.3389/fneur.2015.00025)
Supplement: Supplementary file 1 [file Presentation_1.PDF]

Supplementary Material

EEG spectral features discriminate between Alzheimer's and Vascular dementia

Emanuel Neto[1,4], Elena A. Allen[1,2,3], Harald Aurlien[4], Helge Nordby[1], Tom Eichele[1,2,4]

(1) Institute of biological and medical psychology, University of Bergen, Norway
(2) K.G. Jebsen Center for Research on Neuropsychiatric Disorders
(3) The Mind Research Network, Albuquerque, New Mexico 87106, USA
(4) Section for clinical neurophysiology, Haukeland University Hospital, Bergen, Norway

* **Correspondence:** Emanuel Neto, Institute of biological and medical psychology, University of Bergen, Norway, netoemanuel@gmail.com

Supplementary Tables and Figures

1.1 – Tables

Table 1 -Prescript of medications at the time of EEG recording using the WHO Anatomical Therapeutic Chemical (ATC) Classification System

ATC group code of Medication	**Controls**	**AD**	**VaD**
A02 DRUGS FOR ACID RELATED DISORDERS	8	2	2
A03 DRUGS FOR FUNCTIONAL GASTROINTESTINAL DISORDERS	1	1	1
A06 DRUGS FOR CONSTIPATION	0	1	0
A07 ANTIDIARRHEALS, INTESTINAL ANTIINFLAMMATORY	0	0	1
A10 DRUGS USED IN DIABETES	8	2	3
A11 VITAMINS	0	1	2
A12 MINERAL SUPPLEMENTS	3	1	3
B01 ANTITHROMBOTIC AGENTS	36	30	33
B03 ANTIANEMIC PREPARATIONS	3	0	2
C01 CARDIAC THERAPY	5	5	7
C02 ANTIHYPERTENSIVES	5	1	1
C03 DIURETICS	3	10	9
C07 BETA BLOCKING AGENTS	12	12	16
C08 CALCIUM CHANNEL BLOCKERS	10	5	11
C09 AGENTS ACTING ON THE RENIN-ANGIOTENSIN SYSTEM	21	18	14
C10 LIPID MODIFYING AGENTS	16	15	17
D05 ANTIPSORIATICS	0	1	0
G03 SEX HORMONES AND MODULATORS OF THE GENITAL SYSTEM	2	2	3
G04 UROLOGICALS	5	1	2
H02 CORTICOSTEROIDS FOR SYSTEMIC USE	4	3	3
H03 THYROID THERAPY	10	6	5
J01 ANTIBACTERIALS FOR SYSTEMIC USE	3	1	1
L01 ANTINEOPLASTIC AGENTS	2	0	1

L02 ENDOCRINE THERAPY	1	0	0
M01 ANTIINFLAMMATORY AND ANTIRHEUMATIC PRODUCTS	7	1	3
M02 TOPICAL PRODUCTS FOR JOINT AND MUSCULAR PAIN	1	1	1
M03 MUSCLE RELAXANTS	0	1	1
M04 ANTIGOUT PREPARATIONS	0	3	1
M05 DRUGS FOR TREATMENT OF BONE DISEASES	1	0	0
N02 ANALGESICS	9	8	6
N03 ANTIEPILEPTICS	11	8	16
N04 ANTI-PARKINSON DRUGS(DOPAMINERGIC AGENTS)	0	1	4
ALL PSYCHOANALEPTICS N05x	8	14	19
N05A ANTIPSYCHOTICS	1	4	10
N05B ANXIOLYTICS	5	8	6
N05C HYPNOTICS AND SEDATIVES	5	6	3
N06A ANTIDEPRESSANTS	5	21	15
N06D ANTI-DEMENTIA DRUGS	0	21	5
R01 NASAL PREPARATIONS	2	0	1
R03 DRUGS FOR OBSTRUCTIVE AIRWAY DISEASES	7	3	3
R05 COUGH AND COLD PREPARATIONS	2	0	0
R06 ANTIHISTAMINES FOR SYSTEMIC USE	0	2	4
S01 OPHTHALMOLOGICALS	0	1	0
Dont remember	0	18	12
Absent information	25	21	16
Others, unspecified or unknown	8	8	23

Table 2 - Selection criteria

Group of patients	Inclusion criteria	Exclusion criteria
Controls (**NC**)	- common criteria*	- Diagnosed with any brain-related disease(i)
Alzheimer patients (**AD**)	- common criteria*<br>**- F00.x** Dementia in Alzheimer's disease(ii)<br>**- G30.x** Alzheimer's dementia senile and presenile forms(iii)	- Other dementias forms(iv)
Vascular dementia (**VaD**)	- common criteria*<br>**- F01** Vascular dementia(v)	- Other dementias forms(iv)

* The common criteria included subjects with ages above 30 years old; that were not hospitalized at the time of the EEG recording; only one dataset (the latest) was considered per subject which date of recording if not before existing diagnose

i) **A80-A89 Certain infectious and parasitic diseases**(Viral and prion infections of the central nervous system)
C70, C71, C79.3, D33, D43 Neoplasms (Malignant or benignant in brain and/or central nervous system)
F00-F99 Mental, Behavioral and Neurodevelopmental disorders
G00-G97 Diseases of the nervous system(Other disorders of the nervous system)
I60-I69 Diseases of the circulatory system (Cerebrovascular diseases)
Q00-Q04 Congenital malformations, deformations and chromosomal abnormalities(Congenital malformations of the nervous system)

ii) **F00.x Dementia in Alzheimer's disease**
F00.0 Dementia in Alzheimer's disease with early onset

F00.1 Dementia in Alzheimer's disease with late onset
F00.2 Dementia in Alzheimer's disease, atypical or mixed type
F00.8 Dementia in Alzheimer's disease, unspecified

iii) G30 Alzheimer's disease
G30.0 Alzheimer's disease with early onset
G30.1 Alzheimer's disease with late onset
G30.8 Other Alzheimer's disease
G30.9 Alzheimer's disease, unspecified

iv) Other types of dementia:
F02 Dementia in other diseases classified elsewhere
F02.0 Dementia in Pick's disease
F02.1 Dementia in Creutzfeldt-Jakob disease
F02.2 Dementia in Huntington's disease
F02.3 Dementia in Parkinson's disease
F02.4 Dementia in human immunodeficiency virus [HIV] disease
F02.8 Dementia in other specified diseases classified elsewhere
F03 Unspecified dementia (Without additional symptoms or with other symptoms such as (predominantly delusional, hallucinatory, depressive or other mixed symptoms)
F06 Other mental disorders due to brain damage and dysfunction and to physical disease
F06.0 Organic hallucinosis
F06.1 Organic catatonic disorder
F06.2 Organic delusional [schizophrenia-like] disorder
F06.3 Organic mood [affective] disorder (organic, bipolar or depressive disorders)
F06.4 Organic anxiety disorder
F06.5 Organic dissociative disorder
F06.6 Organic emotionally labile [asthenic] disorder
F06.7 Mild cognitive disorder associated (or not) with a physical disorder
F06.8 Other specified mental disorders due to brain damage and dysfunction and to physical disease
F06.9 Unspecified mental disorder due to brain damage and dysfunction and to physical disease

v) F01 Vascular dementia
F01.0 Vascular dementia of acute onset
F01.1 Multi-infarct dementia
F01.2 Subcortical vascular dementia
F01.3 Mixed cortical and subcortical vascular dementia
F01.8 Other vascular dementia
F01.9 Vascular dementia, unspecified

1.2 – Figures

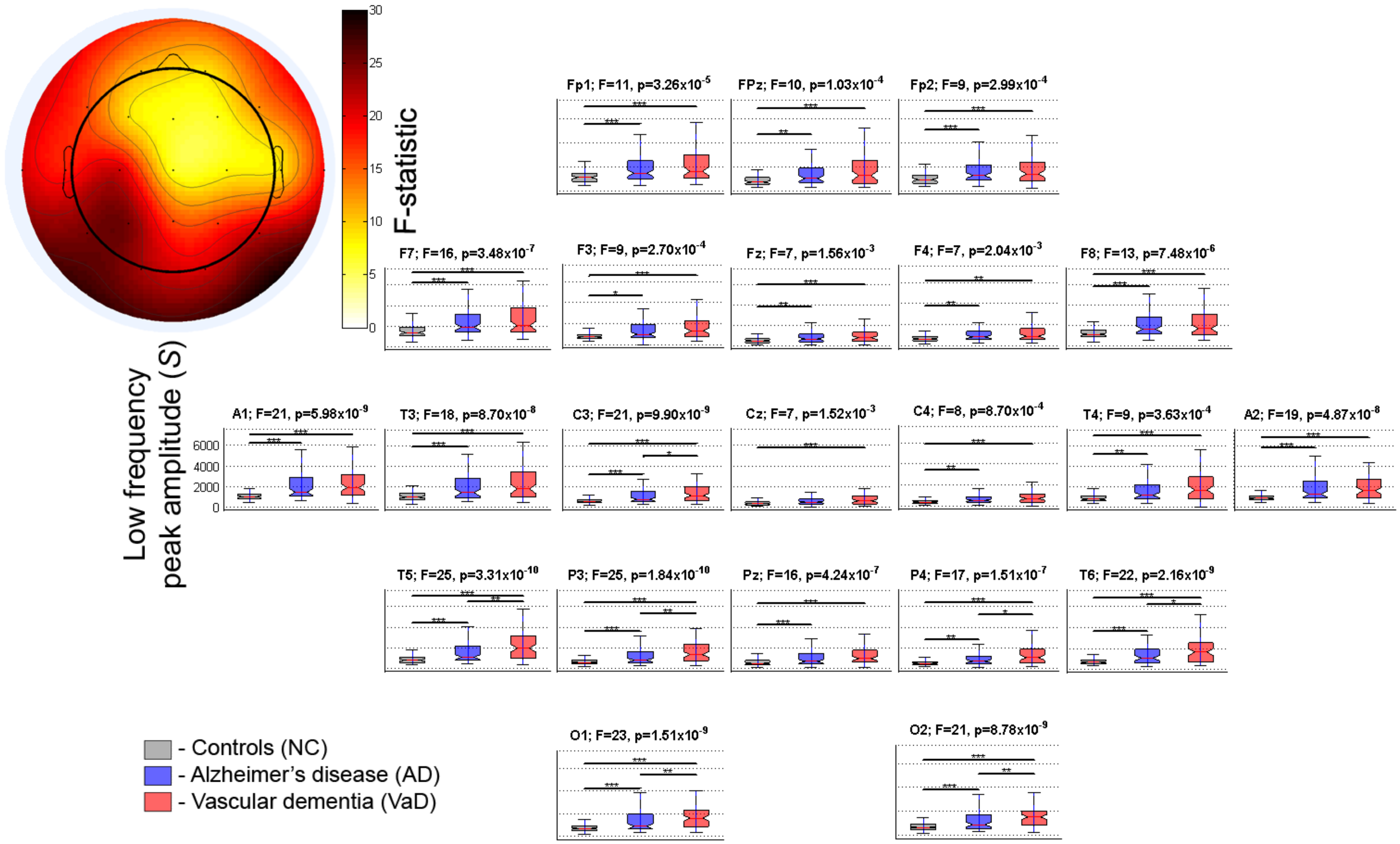

Figure 1: Boxplots depicting spectral parameter *S* for each group at each channel. F-statistics and p-values correspond to one-way ANOVAs performed at each channel and are additionally depicted in the topoplot in the upper left corner. Significant differences between groups (determined with follow-up t-tests) are designated with "*" for $p<0.05$, "**" for $p<0.01$ and "**" for $p<0.001$.

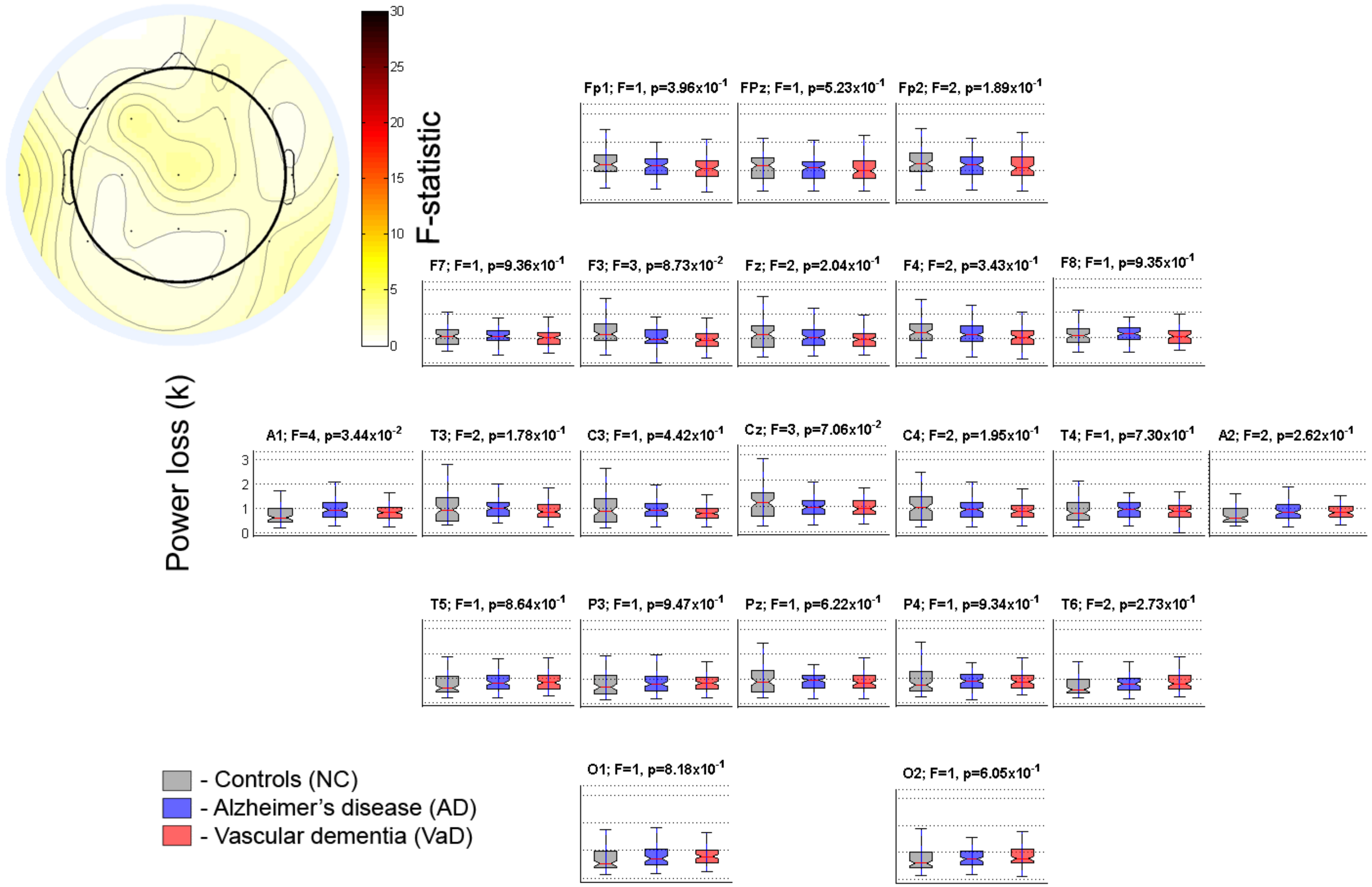

Figure 2: Boxplots depicting spectral parameter *k* for each group at each channel. Display conventions follow those of Figure 1.

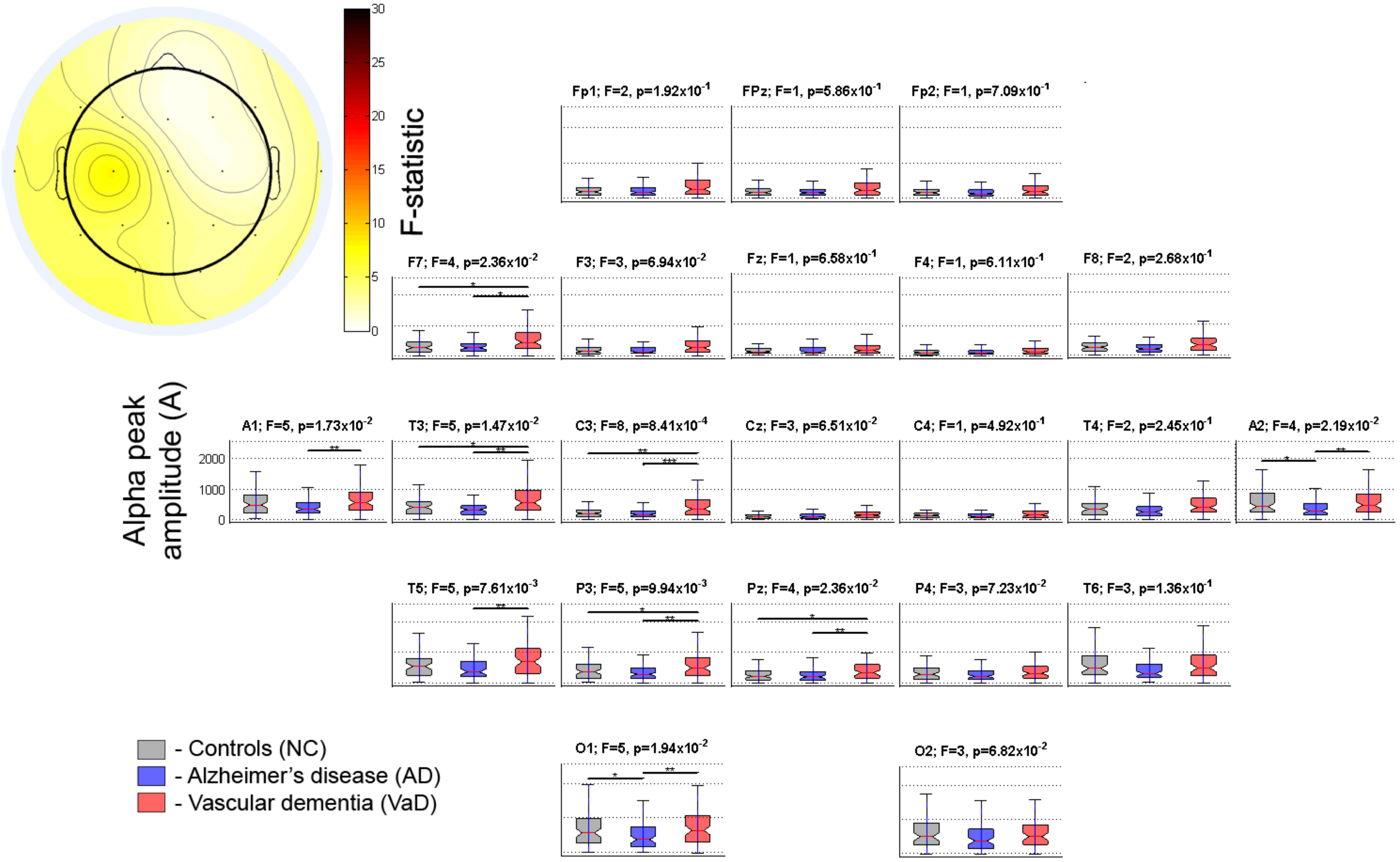

Figure 3: Boxplots depicting spectral parameter *A* for each group at each channel. Display conventions follow those of Figure 1.

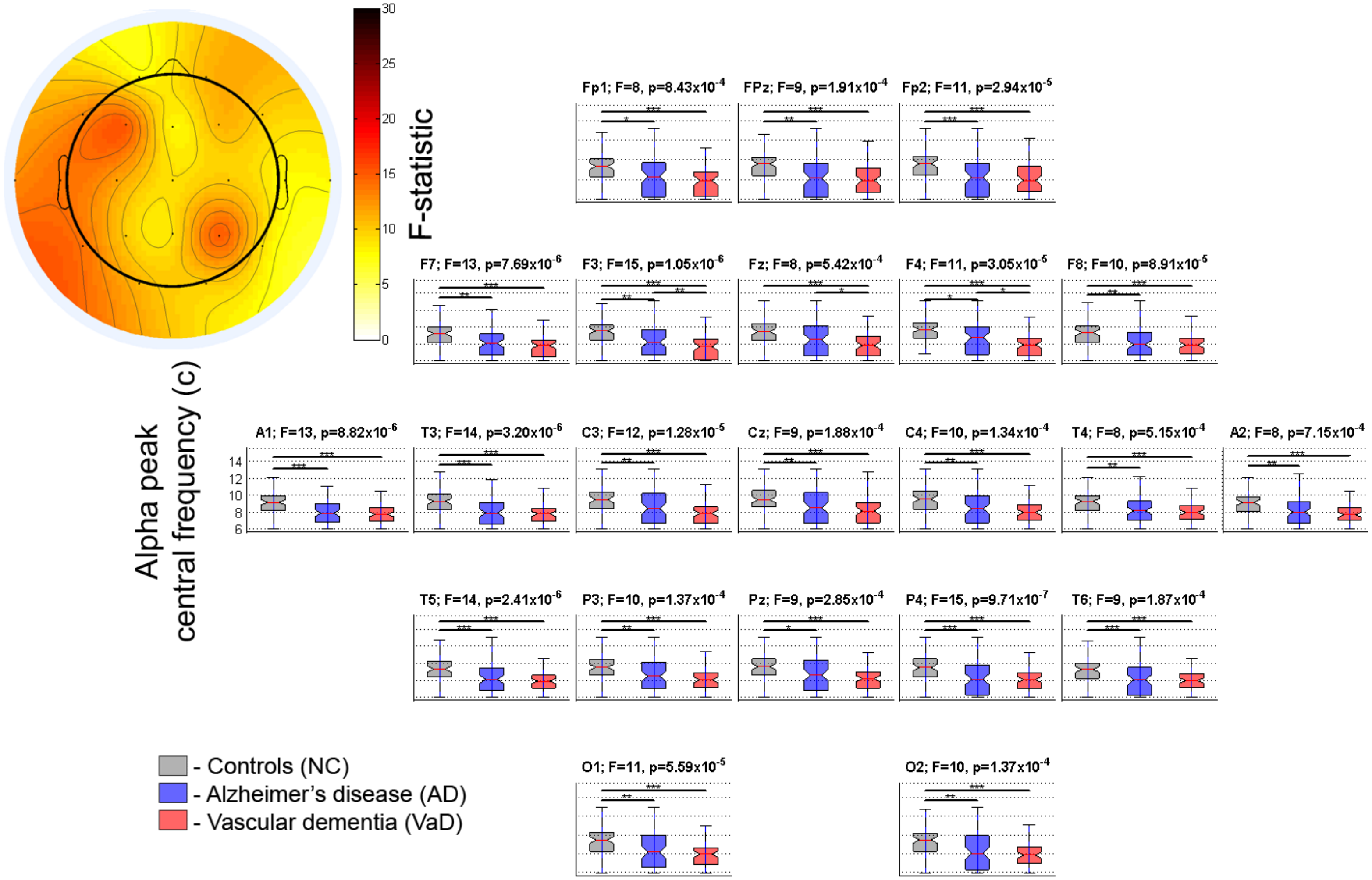

Figure 4: Boxplots depicting spectral parameter *c* for each group at each channel. Display conventions follow those of Figure 1.

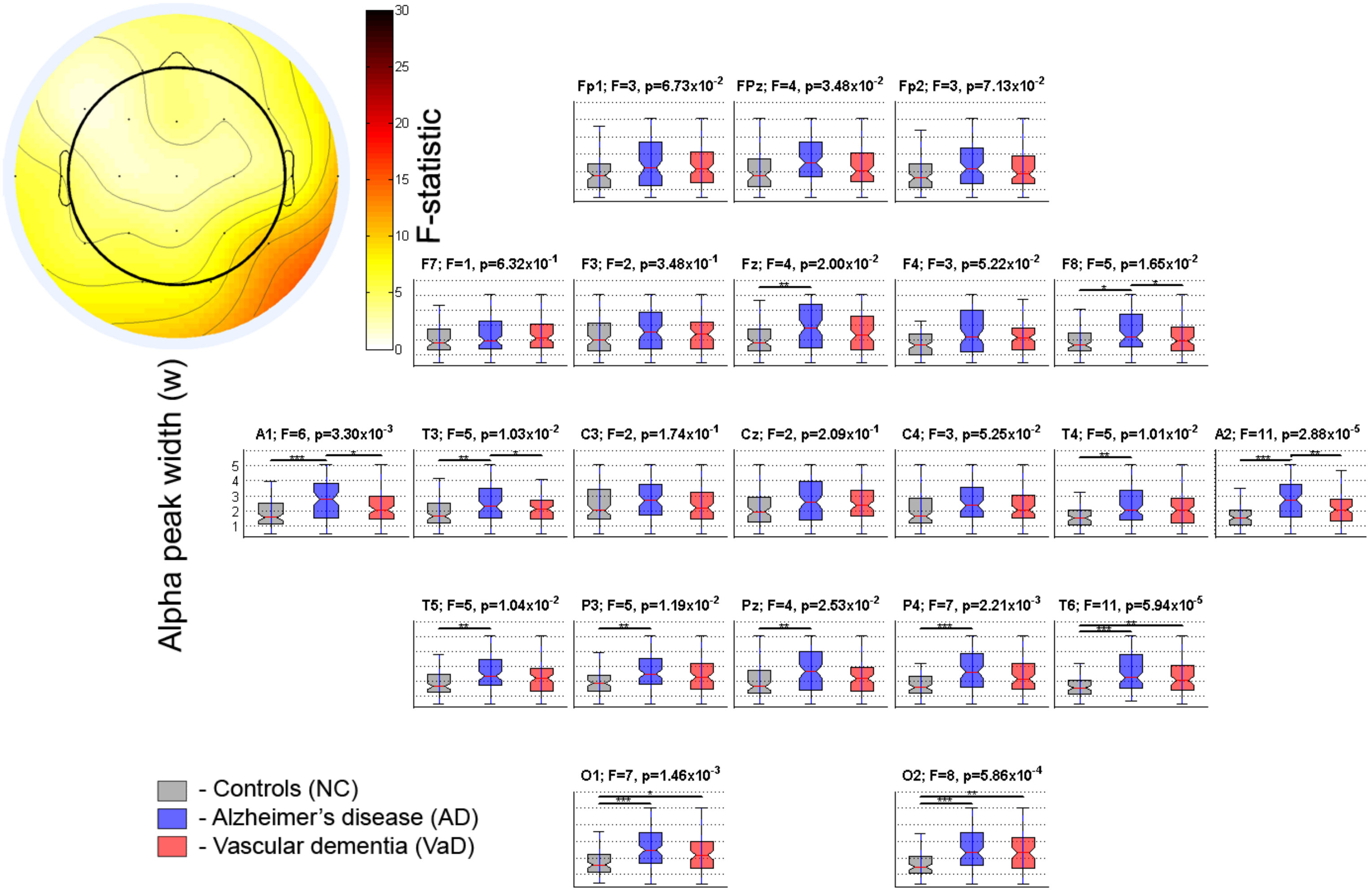

Figure 5: Boxplots depicting spectral parameter *w* for each group at each channel. Display conventions follow those of Figure 1.

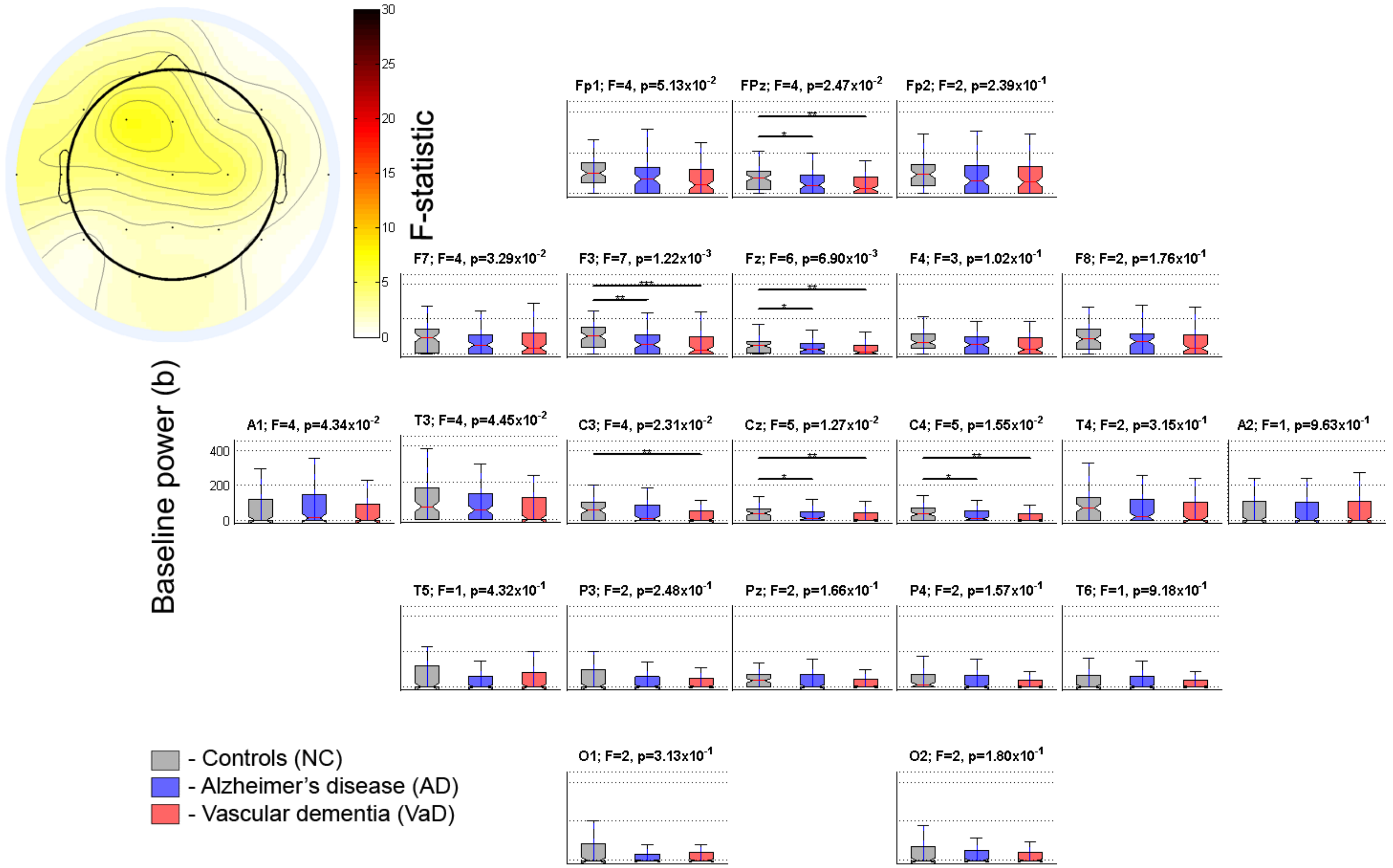

Figure 6: Boxplots depicting spectral parameter *b* for each group at each channel. Display conventions follow those of Figure 1.
